# Porous Inorganic Carriers Based on Silica, Calcium Carbonate and Calcium Phosphate for Controlled/Modulated Drug Delivery: Fresh Outlook and Future Perspectives

**DOI:** 10.3390/pharmaceutics10040167

**Published:** 2018-09-25

**Authors:** Alexey D. Trofimov, Anna A. Ivanova, Mikhail V. Zyuzin, Alexander S. Timin

**Affiliations:** 1 Department of Nanophotonics and Metamaterials, Saint Petersburg National Research University of Information Technologies, ITMO University, 197101 St. Petersburg, Russia; alexeydtrofimov@gmail.com; 2 Research School of Chemical and Biomedical Engineering, National Research Tomsk Polytechnic University, Lenin Avenue 30, 634050 Tomsk, Russia; metallurg_annet@mail.ru; 3 Department of Micro- and Nano-Encapsulation, First Pavlov State Medical University of St. Petersburg, Lev Tolstoy str. 6/8, 197022 Saint-Petersburg, Russia

**Keywords:** drug delivery systems, silica-based particles, calcium carbonate, calcium phosphate, in vitro and in vivo delivery, drug loading

## Abstract

Porous inorganic nanostructured materials are widely used nowadays as drug delivery carriers due to their adventurous features: suitable architecture, large surface area and stability in the biological fluids. Among the different types of inorganic porous materials, silica, calcium carbonate, and calcium phosphate have received significant attention in the last decade. The use of porous inorganic materials as drug carriers for cancer therapy, gene delivery etc. has the potential to improve the life expectancy of the patients affected by the disease. The main goal of this review is to provide general information on the current state of the art of synthesis of the inorganic porous particles based on silica, calcium carbonate and calcium phosphate. Special focus is dedicated to the loading capacity, controllable release of drugs under internal biological stimuli (e.g., pH, redox, enzymes) and external noninvasive stimuli (e.g., light, magnetic field, and ultrasound). Moreover, the diverse compounds to deliver with silica, calcium carbonate and calcium phosphate particles, ranging from the commercial drugs to genetic materials are also discussed.

## 1. Introduction

**Figure 1:**
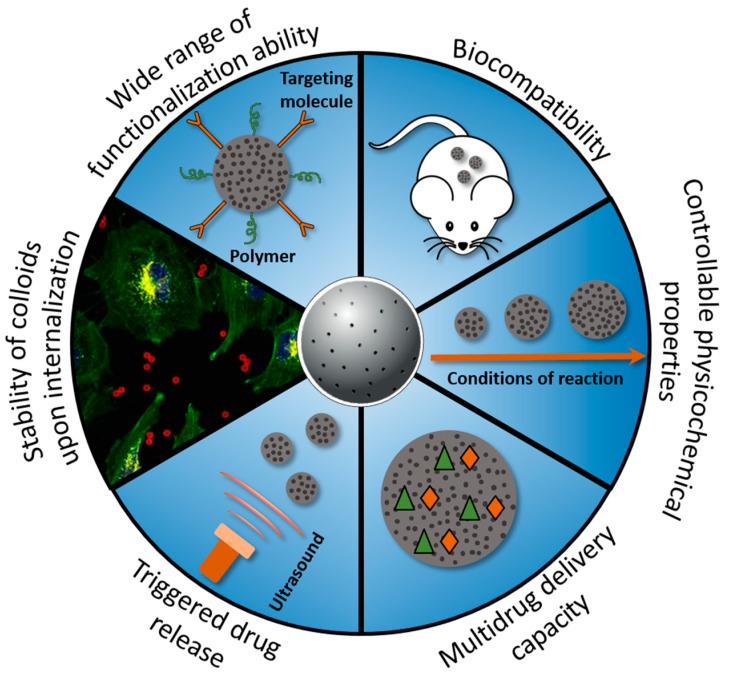
Schematic overview of some advantages of porous carriers for the drug delivery applications.

Nanoscale materials attract a significant interest in the industrial and research communities. These smart functional materials, which are made up of diverse components, offer tremendous opportunities in the field of biomedical applications, especially, in cancer therapy [1,2]. The development of porous materials for the cancer research is mainly determined by the unique ability to control physico-chemical characteristics and properties of these materials [3,4]. At the same time, controllable properties allow to design multifunctional materials, enabling bioimaging, sensing, as well as their therapeutic applications [5]. The porous nano-/microparticles are considered to be the most appropriate carriers for the drug delivery. Among them, silica, calcium carbonate and calcium phosphate are promising vehicles [6,7,8]. These particles are able to transport the huge loads of anticancer or therapeutic bioactive molecules to the desired sites for the disease treatments, owning higher efficiency and lower side effects compared to the individual drugs [9]. The drug carriers based on silica, calcium carbonate and calcium phosphate nano-/microparticles possess several advantages such as improved pharmacokinetic profile, possibility to employ lipophilic drugs, higher circulation time in comparison to the administration of the free drugs and lower overall toxicity due to the lower dosages used [10]. A broad range of the pore sizes (2–50 nm) of porous carriers allows loading of therapeutic agents of different nature, such as low-molecular weight drugs, proteins, genetic materials (plasmid deoxyribonucleic acids (DNA), ribonucleic acids (RNA)). The available surface area and pore networks provide possibility to incorporate even inorganic nanoparticles (for example, iron oxide, gold, silver nanoparticles, quantum dots, etc.) [11,12,13]. The inclusion of the inorganic nanoparticles into the porous structure of the carriers leads to the formation of hybrid materials. These materials combine the properties of both components of the hybrid system (porous carriers and inorganic nanoparticles) [10]. The progress in this field includes the fabrication of porous micro- and nanocarriers modified with, for example, magnetic nanoparticles, what enables the sensitivity of the carriers to the external magnetic field [14]. Combination of various properties in one intelligent carrier provides their multifunctionality for the potential use in the medical diagnostic and cancer therapy. These include in vivo tracking of drug vehicles using magnetic resonance imaging (MRI), application of various field-dependent magnetic behaviors to reach greater concentrations of functionalized drug-particulate systems in the desired areas in vivo. The multifunctional properties can be also achieved when incorporating gold nanoparticles or quantum dots into the porous structure. As known, the coupling of the strong near-infrared plasmon resonance absorption of gold nanoparticles into the thermal energy is an example of a nanoscale phenomenon that has been widely exploited to photothermally destroy malignant tumors. As a result, the gold-functionalized porous carriers are able to deliver antitumor drugs into cancer cells and simultaneously deliberate photothermal properties [15,16]. In addition, the impregnation of inorganic nanoparticles that are intrinsically responsive to different kinds of stimuli allows to form multi-stimuli responsive drug carriers [17]. Employing different types of stimuli (internal or external), a rapid or prolonged release profile can be achieved suggesting multiple drug regimens to improve clinical success [18,19]. Particularly, the multi-functionalized carriers combining two or more stimuli-responsive mechanisms hold greater potential for controlling drug release process and improving therapeutic performance [20]. The surface of porous carriers can be easily functionalized by a variety of ligands such as antibodies, aptamers, bioactive polymers, and peptides for higher in vivo stability in the blood stream (e.g., using PEGylation to avoid macrophage capture, and/or adjusting active targeting properties for the carriers accumulation in the desired area) [21]. Moreover, the size of porous carriers can be tuned using different synthetic approaches. Varying the size and shape of the particles, it is possible to control the internalization process in different cell types [22,23]. In addition, silica, calcium carbonate and calcium phosphate-based carriers are biodegradable and can be safely excreted by the body through the kidney. Figure 1 schematically represents the main advantages of the employment of the porous carriers in the drug delivery applications.

### Scope

Hundreds of review papers devoted to the use of particles as drug delivery carriers have been published in recent years. The majority of review articles dedicated to these materials are focused either on one type of porous particles (e.g., silica, calcium carbonate or calcium phosphate), or on the very general description of porous materials [10,24]. The present review paper aims to provide an overview of three promising types of porous inorganic carriers (silica, calcium carbonate and calcium phosphate). It aims to summarize recent progress in research on the use of porous silica, calcium carbonate and calcium phosphate carriers for the delivery of drugs in vitro and in vivo. This review is not meant to be an exhaustive description of details of either of these technologies or to summarize all existing research on it, but to give a perspective onto these three existing approaches, their advantages, limitations and current state of research progress so that the reader can get a lay of land and plan research accordingly. For each type of carriers there are many comprehensive review papers that can provide an in-depth look into them in general or into a particular aspect of those, be it synthesis, drug loading, drug release or biomedical applications. For the porous materials synthesis (especially, silica), the readers can be also referred to the excellent review articles [25,26]. Additionally, detailed description of the different functionalization strategies for the decoration of nanomaterials with various compounds can be found [27].

This review consists of three sections, not including introduction and conclusions. Each section describes one of the considered porous material (silica, calcium carbonate or calcium phosphate) and is organized as follows: synthesis methods of the porous carriers, drug loading approaches, cargo release, and in vitro/in vivo drug delivery applications.

## 2. Silica-Based Drug Carriers

The porous silica-based carriers can be divided into three types: mesoporous silica nanoparticles (MSNs), mesoporous organosilica nanoparticles (MONs) and periodic mesoporous organosilica (PMO). MSNs category is the most used particulate system [27]. Other two types of silica-based carriers (MONs and PMO) are emerging as new generation of silica hybrid analogs. The advantages of silica-based carriers include well-studied, predictable and low-cost manufacturing process. They are chemically stable what allows them to protect fragile cargo (such as DNA) from the biodegradation before they reach their intended destination. Moreover, silica-based particles are biodegradable at the long term and can be cleared from the organism after several days [1]. In the previous study, it has been demonstrated that the clearance rate of MSNs is primarily dependent on the particle shape. Short-rod shaped silica nanoparticles showed faster clearance rate compared with the long-rod shaped MSNs [28]. Another study indicated hepatobiliary clearance of silica nanoparticles observed over a period of 15 days in vivo [29]. The surface properties of MSNs play also significant role in in vivo excretion. He et al. showed that differently coated silica particles (OH–SiNPs, COOH–SiNPs, and polyethylene glycol (PEG)–SiNPs) were cleared from the systemic blood circulation, but the clearance time and subsequent biological organ deposition were dependent on the surface chemical modification of the MSNs [30].

### 2.1. Preparation Methods and Physical Chemistry of Silica-Based Carriers

MSNs, MONs, and PMO are commonly fabricated using sol-gel processes in aqueous solutions [9,31,32,33]. The conventional sol-gel synthesis has been studied extensively and allow precise control of MSNs properties: size, pore size and geometry, particle modification or surface functionalization [31]. In the typical sol-gel synthesis, silica particles are formed via hydrolysis of various silanes and/or silicates with a subsequent silica condensation:

–Si–O– + HO–Si– → –Si–O–Si– + OH–

Synthesis process takes place in an aqueous solution and may involve alcohol and ammonia or other catalyst [32]. The synthesis reaction speed depends on the pH value with the maximum silica condensation rate at normal pH conditions. Types and concentrations of the used reagents affect the resulting particle size. Tetraethyl orthosilicate (TEOS), tetramethyl orthosilicate (TMOS) and other compounds can be used as silicon sources. Using TMOS facilitates the generation of monodispersed particles since it reacts preferentially with the silanol groups on the surface of already formed particles rather than with itself to create the new particles [33]. To inhibit silica growth and, thus, obtain smaller MSNs, surface-protection agents can be used, such as triethanolamine (TEA), poly (ethylene glycol) (PEG) or a second nonionic surfactant [34]. These agents are also useful for the isolation of the growing silica particles from each other, preventing their aggregation and growth of silica bridges between neighboring particles.

To obtain MSNs, micelles are often used as a soft template to form the mesoporous structure. The silica particles are grown on the templates as starting points for the condensation. Surfactants such as cetyltrimethylammonium bromide (CTAB) are usually added to the solution as well. At low concentrations just above the critical micellar concentration, the surfactant molecules bind together and form small spherical micelles. At higher concentrations, micelles can have cylindrical or other shapes. These micelles are positively charged and attract negatively charged silanes, facilitating their condensation. Addition of the second surfactant can lead to the formation of the more complicated micellar structures, allowing further modification of the MSNs pore structure. Similar to the micelles, vesicles can be used as templates for the MSN growth [35]. Inorganic nanoparticles, such as metal (Au, Pt) or metal oxide (Fe_3_O_4_) nanoparticles could be incorporated into the structure of MSNs [36,37,38]. They can be used as the templates for the MSNs growth as well. So synthesized hybrid particles can be capable of both carrying a drug load and being contrast agents for bioimaging, for example.

Some applications require larger pore sizes to accommodate higher quantities of molecules or simply larger biomolecules, such as DNA and proteins. Several swelling agents can be used to increase the pore sizes, e.g., trimethylbenzene (TMB) [39]. Another way to increase the size of the pores is the use of the block-polymers as templates [40].

After the growth, the templates need to be removed from the MSNs in a way that does not damage the particles. Due to the high thermal stability of silica, simple calcination process can eliminate the most organic templates from the MSNs. However, drying causes MSNs to get into close contact with each other and develop bonds by the dehydration of the surface silanol groups, thus damaging them. Therefore, simple calcination is not the preferable method of template removal. A so-called “liquid calcination” method has been developed using high boiling solvents to retain liquid phase during calcination. Silanol groups can also be removed from MSN surface via a silane ethanol solution, reducing the bridging. The calcination process could be avoided entirely if templating is done using a thermosensitive polymer (poly (*N*-isopropylacrylamide)), which forms aggregates at higher temperatures and dissolves at lower temperatures [41].

The mixture of silane [usually tetraethyl orthosilicate(TEOS)] and an organosilane induces the formation of MONs and PMO. In this case, the surfactant templates should be removed with less aggressive extraction procedures, in order not to destroy the inorganic-organic framework of MONs and PMO. The calcination procedure, which is usually used for MSNs, is not completely appropriate for MONs and PMO. In general, harsh pH and temperature conditions are usually employed for the extracting process. The silica-etching chemistry [alkaline or hydrofluoric acid(HF) etching] is introduced into the synthesis to form the hollow PMO structure [42]. For this, the PMO layer is directly deposited onto the surface of silica particles in order to form well-defined solid silica core/PMO shell. The chemical stability of some families of PMOs is higher than for the silica particles under etching. Therefore, the silica core can be selectively removed under alkaline or HF etching producing hollow periodic mesoporous structure. For instance, Wu et al. have successfully developed the strategy for the fabrication of hollow-structured mesoporous silica particles with the large pore sizes and the organic-inorganic hybrid frameworks [2]. Another example of the fabrication of hollow periodic MONs was demonstrated by Chen et al. [43].

**Figure 2:**
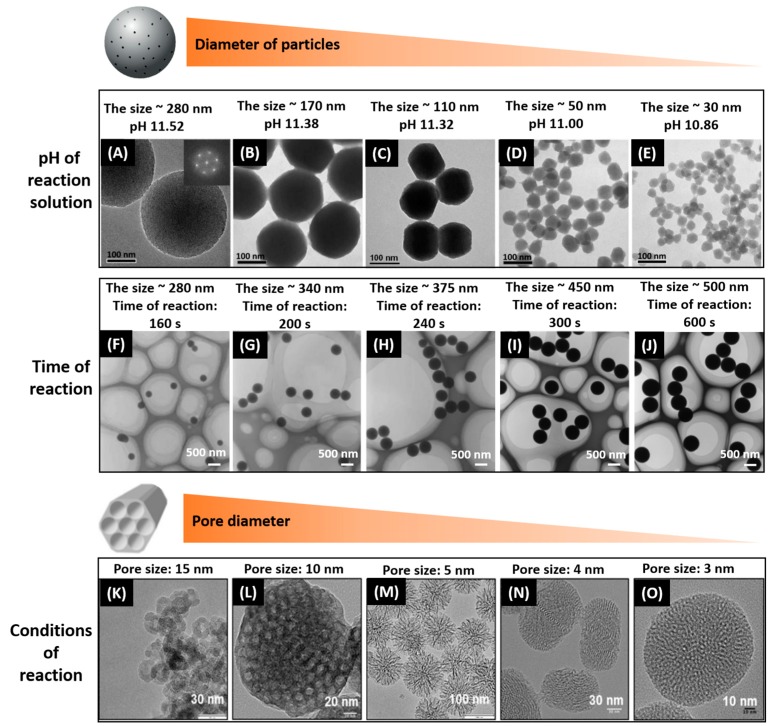
TEM images of silica-based carriers with tunable diameters and pore size. (**A**–**E**) Reproduced with permission from [31]. Royal Society of Chemistry, 2013; (**F**–**J**) Reproduced with permission from [33]. American Chemical Society, 2007; (**K**–**O**) Reproduced with permission from [45]. American Chemical Society, 2017.

The high attention on silica-based carriers can be associated with their versatility in size, morphology, and texture. The uniform silica particles of different diameters can be prepared using various synthetic conditions (e.g., controlling pH values or time of reaction) (Figure 2A,B). For instance, Fang Lu et al. [44] developed a simple method for tailoring the size of well-ordered and dispersed MSNs by adjusting the pH of the reaction medium, which leads to the series of MSNs with diameter sizes ranging from 30 to 280 nm (Figure 2A). It also possible to control particle growth at different times of the reaction (Figure 2B). Smaller particles (140 nm) emerged for 160 s into the reaction process grew to their final size (500 nm) in 600 s. Optimizing the particle size is highly important for the biomedical applications, since the size, shape and morphology of particles can significantly influence cell uptake, accumulation inside tissues and organelles.

Since the efficiency of drug loading and its further release depends on the pore structure and composition, it is very important to control the pore parameters for the developing of drug carriers with the optimal pore sizes. Sol-gel chemistry is the most suitable method to obtain highly ordered silica-based materials with different pore sizes. By varying conditions of sol-gel synthesis through templating and pore-swelling agents, different porous silica-based materials can be prepared, which define the structural diversity of the silica-based carriers. The small-pore MSNs with radially disposed disordered pores (about 3 nm) are shown in Figure 2C. These materials can be prepared using a procedure where the common base NaOH was replaced by the polyalcohol triethanolamine (TEA). Thus, this allows conducting the synthesis at a lower pH values, while the condensation is influenced by the chelating properties of the TEA. By combining these features with the specific time- and temperature-profiles, discrete mesoporous nanoparticles with narrow particle size distributions can be prepared [34]. The synthesis of MSNs with ordered pores of about 4 nm with a helical arrangement and an amino-functionalized MSNs with expanded stellate pores of about 5 nm can be synthesized in the basic reaction solutions [45]. It is also possible to fabricate the MSNs with the bottleneck pores of about 10 nm (large pore MSN or LP-MSN) and single pore MSNs with even larger pore diameters (15 nm) using an adapted acidic synthesis strategy, which was described in the Ref. [46]. Thus, there are many possibilities available for tuning structure and functionality of MSNs by means of synthetic strategies. At the same time, the composition of the mesoporous walls can be modified with organic moieties, which allow manipulating the loading efficiency of drugs, as well as their release behavior upon internal and external triggering stimuli [45].

### 2.2. Drug Loading Approaches

There are different approaches to load the cargo into the silica-based carriers. The incorporation of drugs into the pores of silica-based materials is widely reported. MSNs can be loaded with hydrophobic and hydrophilic drugs. The hydrophobic drugs can be loaded via noncovalent pore-drug interactions, such as electrostatic bonding between negatively charge silica pores and positively charge functional groups of drugs (e.g., doxorubicin, vincristine). The main disadvantage of this drug loading approach is that open pores leave the drug free to interact with the surroundings, allowing leakage and degradation. To overcome this problem, several loading strategies have been developed. The loading of diverse therapeutic small-sized compounds can be performed using the outer surface engineered approach, when the surface of MSNs are functionalized with polymers, proteins, lipids, or nanoparticles. The polymer coating block the diffusion of the loaded drugs [3,4,47,48]. Moreover, the grafting of nanoparticles (a few nanometers in diameter) onto silica surface allows incorporating drugs. As an example, Lin and colleagues reported the use of the gold nanospheres [49], iron oxide nanospheres [50] etc. The loading of drugs could be achieved by change of the protein conformation or electrostatic interactions between protein and drug [48,51]. Besides outer surface engineered approach, the pores of silica can be functionalized with organic groups of organosilane to enhance the drug loading efficiency [4,51]. The organic functional groups of MONs can interact with active drugs via electrostatic forces, chemical bonding, or noncovalent interactions. For instance, Lin and co-workers developed MSNs with amino-coumarin functional groups, which can covalently link to the anticancer drug chlorambucil [52]. The uniquely hybridized structure and composition of PMO carriers allows enhancing the loading capacity [49]. The hybridized pore walls of PMO carriers provide strong noncovalent pore-drug interactions and possess a tunable hydrophobicity/hydrophilicity. As a result, many drugs can be loaded in exceptionally high contests into PMO carriers without leakage even in the absence of pore capping agents [53].

### 2.3. Release Efficiency

**Figure 3:**
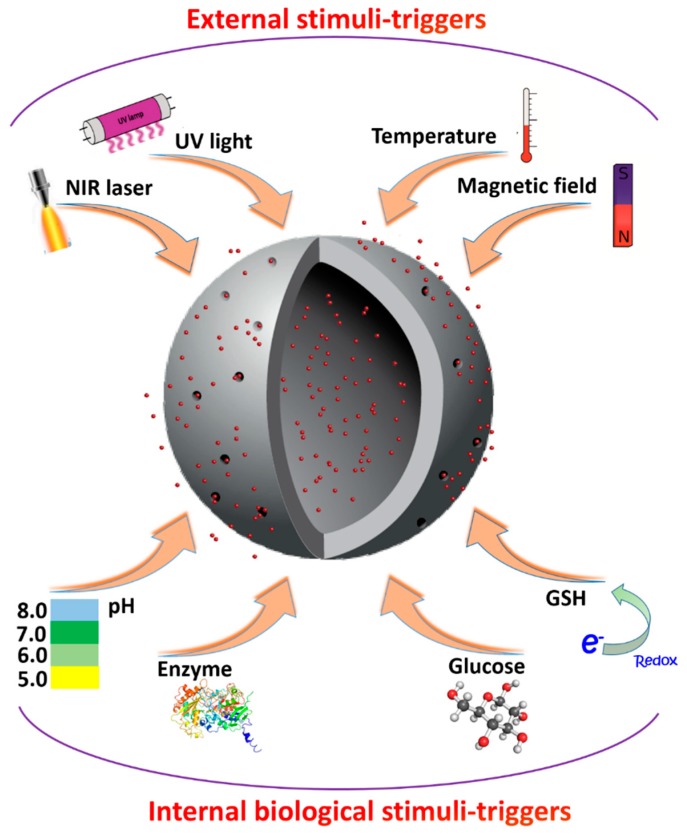
Schematic presentation of a variety of external and internal triggering mechanisms for delivery of bioactive compounds using porous silica-based carriers.

The mechanism of drug loading is closely connected with its release, thus the desired conditions of cargo release determine the coating method. Two distinct mechanisms of payload release can be distinguished: triggering from internal factors, in this case the release occurs when MSN environment reaches certain predetermined conditions, and triggering from external factors, when an outside influence is required for the release to occur (Figure 3). With an internal triggering, release conditions can be chosen close to the conditions inside or near cancerous cells, for example, to deliver chemotherapeutic drugs such as doxorubicin to their targets. With an external triggering, it is possible to select the area for the drug release by applying, for example, light or magnetic field locally.

#### 2.3.1. Internal Triggering

pH trigger: The pH values of surrounding environment can trigger the release of cargo from silica-based carriers. Warburg effect states that pH value is reduced near cancerous cells due to their energy metabolism. The pH value drops even further in endo/lysosomal environment. This can be used to deliver bioactive compounds with further release in acidic conditions inside cells. The loaded cargo can be protected from the leakage in acidic environment by capping the MSN pores with pH-labile bonds, such as acetal or amine bonds, boronate or hydrazine groups. These bonds can hold the cargo inside the pores in a neutral or alkaline environment; however, they will be hydrolyzed in an acidic environment, releasing the cargo. For example, several groups have demonstrated pH-responsive release of doxorubicin from MSNs covered by poly (acrylic acid), poly (styrene sulfonate) or other polyelectrolyte multilayers [51,52,54]. Coating MSNs with a cell-penetrating peptide (deca-lysine sequence K10) and capping them with ZnO nanoparticles was shown to have “zero-premature” drug release in the physiological environment and rapid release inside cancerous cells’ endosomes [55]. Other possibilities of pH-triggered release mechanisms have been tried, including pH-degradable calcium phosphate coating [56] and poly (l-histidine) coating [57].

Redox trigger: Redox triggering is based on the difference in glutathione (GSH) concentration inside intracellular and extracellular environment. The ability of GSH to reduce disulfide bonds is often used for triggering cargo release. GSH concentration inside cells is several orders of magnitude higher, which allows the cargo to be very well-contained before it is internalized by a cell and released afterwards [58]. Moreover, the GSH concentration is considerably higher in cancerous cells due to their higher metabolic rate in comparison with normal cells. Thus, linking the cargo to the MSNs via disulfide bonds will lead to the release of the cargo in the intracellular environment. Alternatively, MSNs with loaded cargo can be capped by macromolecules linked via GSH-sensitive bonds. Various molecules have been studied for this, including β-cyclodextrin [59], peptides [60], hyaluronic acid [61], PEG and other polymers [62,63,64]. Besides capping the pores, these macromolecules can act as targeting agents for the MSNs or serve for other purposes, raising the efficiency of the drug delivery system as a whole. Moreover, disulfide bonds can be introduced directly into MSNs, so that GSH would cleave the MSNs themselves into smaller pieces after delivery, which would also increase their biodegradability and ensure better clearance [65,66].

Enzyme trigger: Enzymes play crucial role in the cell metabolism. Their activity is complicated and enzymes expression levels can vary a lot between different types of cancerous and normal cells. Careful choose of the capping molecule enables the MSN opening after the contact with the certain enzymes. Enzyme triggering can be very efficient, for this, the capping molecules should have bonds that can be degraded by enzymes which are overexpressed in the cancer cells [67]. Therefore, various studies have been performed where anticancer drugs were loaded into MSNs sensitive to the tumor enzymes [64].

#### 2.3.2. External Triggering

Photochemical trigger. Light is a very convenient external trigger for several reasons. It can be easily localized in the treatment area, can be applied noninvasively, as well as delivered inside body cavities via waveguides, affecting only specific compounds tied to a specific wavelength [52]. It has been widely used for targeting tumors in, e.g., photodynamic therapy. There are several photosensitive groups that can be cleaved by light of specific wavelength. For example, o-nitrobenzyl ester group can be cleaved by 350 nm UV light. It was used to cap MSNs with cyclodextrine or other groups to be opened remotely [65,66]. Another example is coumarin that was used to seal a model dye inside MSNs with β-cyclodextrin using two-photon excitation [68]. Doxorubicin was loaded into MSNs capped with coumarin-based block copolymer that was degraded by two-photon infrared (IR) irradiation [69]. Two-photon processes are beneficial in biomedical applications because they allow more precise localized effects. Another photochemical reaction that can be used to trigger the cargo release from MSNs is photoisomerization, when the capping is not cleaved, but “opened” by changing its shape. For example, azobenzene can be switched between its cis and trans forms when exposed to ultraviolet (UV) and visible light, respectively. Azobenzene and β-cyclodextrin were used in creating valves to load or release the model drug doxorubicin inside the MSNs [70].

Thermal trigger. While cancerous tissues usually have slightly elevated temperatures, which can be used for internal triggering, external heating can result in considerably higher temperature variations. Heating can be done either directly or indirectly, by light or alternating (AC) magnetic field. When metal plasmonic nanoparticles embedded into MSNs are irradiated by light, efficient plasmonic heating may occur via light absorption. By using laser pulses of short duration (picosecond or femtosecond) MSNs can be heated to high temperatures while keeping the heating localized near the MSNs, since heat does not have enough time to spread around and damage surrounding area for the pulse duration. As an example, paraffin has been used to seal the cargo inside the MSN. After heating the MSN above the paraffin melting point, it opened and released the cargo [71]. Coiled-coil peptides were also used as MSN cappings [72]. They remain in coiled state while the temperature is below 80 °C, sealing the MSN pores; when the temperature rises above, the coiled-coils disassemble and open the pores. Several groups have used poly-*N*-isopropylacrylamide (PNIPAAm) as a thermoresponsive capping since it swells via absorbing water and shrinks via dehydration at elevated temperatures [69].

Magnetic trigger. Magnetic fields are used to open MSN pores indirectly. First, embedding magnetic nanoparticles inside the MSNs allows their heating by applying AC magnetic field. These MSNs can then be opened via thermal-triggered mechanism and cargo, e.g., doxorubicin [73], can be delivered. AC magnetic field could also induce a mechanical effect and “squeeze” the cargo out of MSN pores. A considerable advantage of magnetic fields is that they produce little to no harmful effect on the organism by themselves, even in comparison with light. Magnetic nanoparticles used in this approach can also be used as contrast agents in magnetic resonance image (MRI), helping with both visualizing and healing [71].

### 2.4. Delivery of Various Compounds In Vitro and In Vivo 

The aforementioned methods of loading and controlled/modulated release of drugs have been used in many studies to deliver a variety of cargo, including model drugs and fluorescent dyes (Table 1). For instance, Wang et al. [74] have developed a pH-triggered drug delivery system by layer-by-layer assembly of chitosan (CHI)/dialdehyde starch (DAS) polyelectrolyte multilayers (PEM) onto MSNs. The formed carriers were used to deliver doxorubicin by changing pH values in vitro. In neutral environment the release of cargo was shown to be slow, whereas at pH 5 the breakage of C=N bonds of the CHI/DAS multilayers resulted in rapid release of doxorubicin. To improve the escape of delivered cargo from the endo/lysosomal compartments after endocytosis, Zhang et al. [61] coated MSNs with a kind of cell-penetrating peptide, deca-lysine sequence (K10). Additionally, ZnO quantum dots were used as capping agents, which are able to dissolve in acidic environment. Synthesized multifunctional carriers were employed in vitro and showed synergistic antitumor effect, as well as preventing premature drug release from MSNs. In vivo studies have been performed as well. For instance, Zhang et al. demonstrated improved uptake of functionalized with folate ligands silica nanoparticles into tumor, as well as delivery of doxorubicin and subsequent inhibition of the tumor growth in mice [75]. Chemotherapeutic drug doxorubicin was also co-delivered with antiangiogenic agent combretastatin A4 with porous silica particles in vivo in the work of Li [76]. The release of combretatatin A4 resulted in disruption of vascular structure, in addition to the chemotherapeutic effect of doxorubicin on tumor.

Ze-Yong et al. also delivered doxorubicin using silica particles in vitro. Fabricated particles were glutathione (GSH) responsive, since the surface of the MSNs was modified with arginylglycylaspartic acid (RGD) peptide using disuldife bonds. As a result, almost no drug leakage from the carrier, as well as efficient receptor mediated endocytosis was observed [60]. The improved study of biodegradability of GSH responsive MSNs loaded with doxorubicin was reported by Wang et al. [77] After analysis, it was demonstrated that after interaction with GSH, carriers were broken into small pieces. Moreover, the negligible hemolytic activity and low cytotoxicity show the excellent biocompatibility of studied particles in vitro. Zhang et al. conjugated hyaluronic acid (HA) with the surface of the MSNs. In this case, HA served not only as a GSH-responsive capping agent capping, but also as a targeting agent, significantly increasing cellular uptake in HeLa cells in comparison to human hepatocyte cells (LO2) cells. Doxorubicin hydrochloride was successfully delivered with the synthesized multifunctional carriers in vitro [61].

Previous studies demonstrated successful delivery of nucleic acid with porous silica nanoparticles. Wang and colleagues delivered messenger RNA (mRNA) loaded into the silica-based nanoparticles pores in vivo [78]. Delivery of nucleic acids and, thus, cell transfection, with silica carriers can be improved by functionalizing of nanoparticles with polyethyleneimine (PEI). However, this can significantly reduce safety profile of porous silica nanoparticles [79]. 

As it has been mentioned, controllable properties, biocompatibility and biodegradability makes MSNs a great candidate for clinical applications. Silica as a material has been generally considered safe for a long time which should help MSNs get regulatory approval. Unfortunately, there have not been many clinical trials involving MSNs yet [80]. A couple of trials involving silica-gold nanoparticle have taken place [80]. They were used not to deliver drugs, but to induce plasmonic heating via IR irradiation [81]. There was, however, one successful clinical trial of so-called Cornell dots, which used silica hybrid nanoparticles to deliver cyanine (Cy5) dye and iodine (^124^I) to facilitate Positron Emission Tomography imaging [82]. The nanoparticles were also modified with cyclic arginine–glycine–aspartic acid (cRGDY) peptides for molecular targeting. The success of the trial proves the potential of silica nanoparticles and paves the way for further clinical evaluation of various MSN-based drug delivery systems.

However, some previous studies in vitro and in vivo showed toxicity and certain hazard effects using MSNs. These adverse effects of MSNs depended on the cell type and physico-chemical properties of MSNs (e.g., size, shape, chemical composition). For example, silica-based carriers may cause the generation of oxidative stress in cells via formation of reactive oxygen species (ROS) [83]. Moreover, it has been shown that it can contribute to the decreasing of glutathione level in cells [84], as well as in the induction of antioxidant enzymes [85]. All above mentioned effects can cause the cell membrane damage. Some works reported that the size of silica particles can play a crucial role in the particles toxicity. Cho et al. demonstrated that bigger silica particles (100 and 200 nm) induced inflammatory response of the liver in vivo, whereas smaller silica particles (50 nm) not [86]. Thus, before introduction silica particles in preclinical/clinical studies, it is important to investigate potential toxicity of these carriers on cells and tissue.

## 3. Calcium Carbonate Drug Carriers

**Figure 4:**
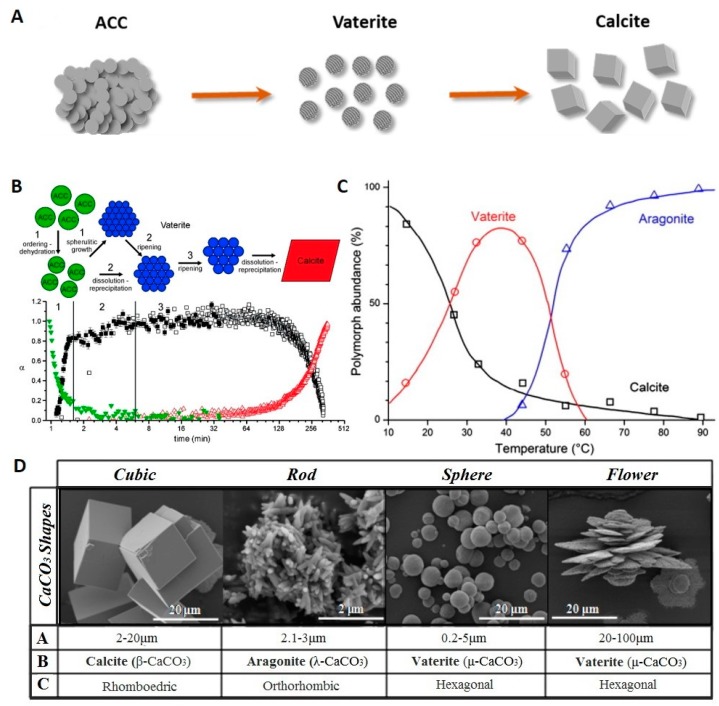
(**A**) Schematic illustration of CaCO_3_ crystallization process; (**B**) Schematic representation of ACC-vaterite-calcite crystallization pathway for the full crystallization reaction in the pure ACC system (the green triangles and full black squares represent the ACC and vaterite from this study, and the open squares and red triangles represent the vaterite and calcite). Reproduced with permission from [92]. American Chemical Society, 2012; (**C**) initial polymorphic composition of CaCO_3_ in dependence of the temperature. Reproduced with permission from [94]. Elsevier, 2014; (**D**) the typical shapes of calcium carbonate (CaCO_3_) particles. (A: Average Diameter; B: Crystalline Phase; C: Crystalline System). Reproduced with permission from [97]. MedCrave, 2017.

Apart from the porous silica-based carriers, porous calcium carbonate (CaCO_3_) has been studied as a potential candidate for the encapsulation of different drugs for decades [89,90,91]. CaCO_3_ naturally presents as calcite, aragonite and vaterite forms. The crystallization of CaCO_3_ polymorphs occurs through the formation and subsequent transformation of amorphous calcium carbonate (ACC) by following pathway (ACC-vaterite-calcite) depicted in Figure 4A,B. As it can be seen, spherulitic growth of vaterite crystals via ACC dissolution is replaced by an Ostwald ripening process of crystal growth. The further ripening of the vaterite is easily determined by a dissolution-precipitation process, which leads to the final calcite [92]. CaCO_3_ polymorph transformation is governed by temperature (Figure 4C) which can be used to prepare particles of a certain phase composition [93]. Calcite and aragonite polymorphs have faceted crystals, whereas vaterite particles are usually spherical or irregular as shown in Figure 4D. Among the polymorph modifications of calcium carbonate, the metastable vaterite is the most practically important because it has large porosity, large surface area, and can be dissolved rapidly under relatively mild conditions. Recently, vaterite particles were considered to be an excellent drug delivery carriers as well as good bioactive material due to its low thermodynamic stability and high solubility [94]. Porous vaterite nano/microparticles offer a large surface area for adsorption of compounds of interest and a possibility of their controlled release.

### 3.1. Synthesis and Loading of Drugs

The existing synthesis methods for obtaining CaCO_3_ nano/microparticles can be divided into two categories: chemical [94,95,96,97] and microbiological [98,99,100,101]. The latter approach is a bacteria-mediated synthesis, which employs products of microbial metabolism containing carbonate ions which react with the calcium ions present in the environment to form CaCO_3_ [97]. Chemical methods are generally based on the emulsion technique [102,103] and the precipitation reaction [104]. The conventional approach for the industrial production of CaCO_3_ is precipitation by carbon dioxide bubbling through a calcium containing solution (gas diffusion method) [104,105]. Moreover, chemical methods include the controlled double-jet precipitation technique [106,107,108] and solvothermal growth [109].

**Figure 5:**
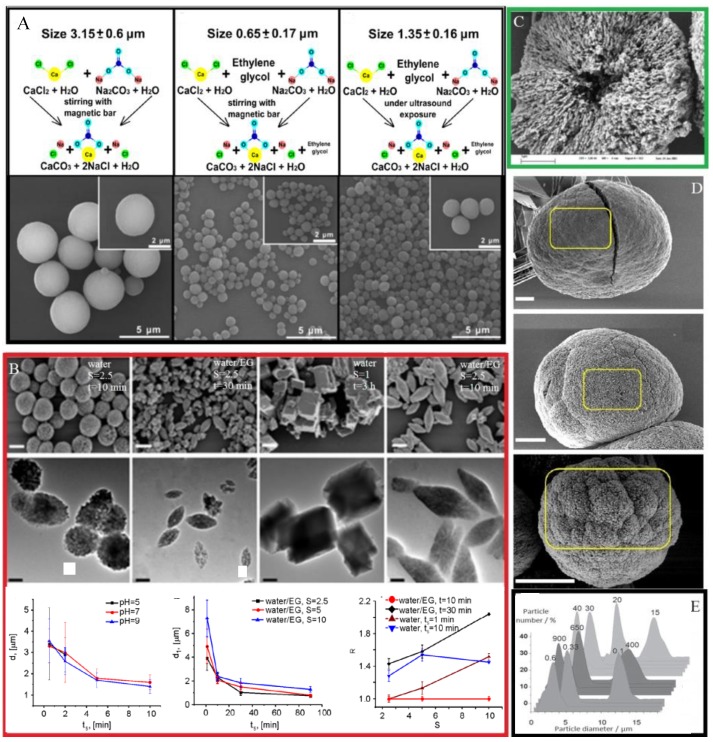
(**A**) Schematic presentation of the three types of synthesis with varied parameters as stirring time, the presence/absence of ethylene glycol, the salt ratio S, and the pH of the solutions and scanning electron microscopy (SEM) images for the obtained vaterite particles with size diameters of 3.15, 0.65, and 1.35 µm vaterite. Reproduced with permission from [122]. Frontiers Media S.A., 2018; (**B**) SEM images of the CaCO_3_ particles synthesized with varying stirring time and presence/absence of ethylene glycol and at pH values of 5, 7, 9 and plots depicted the dependence of particle size from the stirring time. Reproduced with permission from [96]. Springer, 2015; (**C**) SEM images of cross-section of CaCO_3_ microparticle. Reproduced with permission [113]. Royal Society of Chemistry, 2004; (**D**) SEM images of CaCO_3_ particle grown at different temperature. The pore sizes were found to be 19 ± 5, 28 ± 9, and 44 ± 13 nm for crystals prepared at 7.5, 22, and 45 °C, respectively. Reproduced with permission from [109]. American Chemical Society, 2016; (**E**) CaCO_3_ particle size distribution as a function of preparation conditions (salt concentration (*c*), speed (*v*) and time (*t*) of salt stirring). Parameter variations are on the top of the curves. Orange—salt concentration varied (*v* = 650 rpm, *t* = 30 s); violet—stirring speed is varied (*c* = 0.33 M, *t* = 30 s); green—stirring time is varied (*c* = 0.33 M, *v* = 650 rpm). Reproduced with permission from [123]. Wiley Publishing groups, 2012.

The most easiest and most cost-efficient way to synthesize the CaCO_3_ nano/microparticles is spontaneous precipitation by mixing supersaturated solutions of calcium (CaCl_2_ and Ca(NO_3_)_2_) and carbonate salts (Na_2_CO_3_, NaHCO_3_ and (NH_4_)_2_CO_3_) [102,110,111]. In recent years, many efforts have been made to elaborate an in-depth understanding the growth mechanism of CaCO_3_ and to reveal experimental conditions promoting the vaterite particles crystallization. Some of the most impressive results in this field are discussed by Costa et al. and Trushina et al. [94,97]. Concentration of the applied sources of Ca^2+^ and CO_3_^2−^, their type, pH, temperature, mixing speed and the intensity of agitation of the reaction mixture is found to determine the resultant CaCO_3_ polymorph, particle size, morphology and stability of the precipitates [94,96,112,113]. Basically, by changing parameters determining the number of nucleation sites and the crystal nucleation rate the CaCO_3_ properties can be adjusted. For instance, supersaturation is considered to affect the transformation of CaCO_3_ polymorphs during synthesis significantly [114]. Lower nucleation rate delays the formation of more stable polymorphs of aragonite and calcite promoting the vaterite precipitation. pH value of the exploited solution defines its ionic strength. A higher pH leads to a higher concentration of carbonate ions and a higher supersaturation. It was shown that increase of the pH value to 7–11 leads to the precipitation of calcium carbonate in the vaterite modification [115]. Moreover, addition of various inorganic [101,114,116] and organic substances [6,98,103,111,117,118] containing carboxylic, hydroxyl, carboxylate, phosphonate, sulfonate and amino groups induces vaterite particle formation. By changing the concentration of additive, molecular weight and the amount of functional groups the growth rate, crystal habit and stability, particle size and surface morphology can be controlled (Figure 5). Meanwhile, the size and porosity of the CaCO_3_ crystals can be adjusted without any additives simply by changing concentrations of the applied salts, stirring conditions and temperature (Figure 5).

Parakhonskiy et al. studied the influence of CaCO_3_ particle parameters such as the aspect ratio of the particles and their sizes on cell uptake. The authors demonstrated that by controlling physico-chemical parameters of the synthesis such as pH of solutions, the ratio of salt concentrations, and the duration of the reaction the size and shape of the CaCO_3_ particles can be changed [96]. By adjusting the stirring time, the dimension of the particles can be varied at the micrometer range. The geometry of the CaCO_3_ particles (spherical, ellipsoidal and cubical) can be controlled by changing the composition of the applied solvent and salt concentration ratio.

Wang et al. investigated hybrid CaCO_3_ spheres precipitated from the aqueous solution containing carboxymethyl chitosan [119]. Through adjusting the concentrations of Na_2_CO_3_ and CaCl_2_ solutions the micro- and nanospheres can be obtained. The role of bovine serum albumin (BSA) and lysozyme (Lys) in the formation of vaterite is reported in the Ref. [111,120,121]. Thus, synthesis conditions can seriously affect the size (from 20 nm to 10 μm with an average pore size of 20–60 nm) and shape of the obtained vaterite particles; what in its turn determines their final application, drug-loading capacity and cell uptake. However, submicrometric particles are considered to be more favorable for biomedical applications, since these particles can be more effectively internalized by most of cells than their micrometric counterparts. The higher porosity and surface area make vaterite nano/microparticles beneficial for the adsorption of a large variety of drugs and biomolecules.

### 3.2. Drug Loading

There are three approaches to load drug molecules into CaCO_3_ carriers. Active compounds can be co-precipitated during synthesis of the CaCO_3_ [124,125]. Practically, it is applicable for water soluble molecules. Impregnation of the prepared particles in a drug solution under constant stirring or shaking is another approach [111]. In this case, filling of the pores of synthesized cores can be achieved by either physical adsorption of drug molecules or by infiltration. In the latter approach organic solvents can be used which allow loading poorly water-soluble drugs. However, it is challenging to develop an impregnation method with high payload, due to the strong solute-solvent interactions reduce drug adsorption to the CaCO_3_. Therefore, sufficient amount of drugs needs to be dissolved in a solvent to obtain sufficient drug loads. As an alternative to the impregnation approach the solvent evaporation method performed under reduced pressure can be used [126]. This method is applicable for the loading of water soluble and poorly water-soluble drugs. In this case, capillary forces play a role in driving filling of the pores.

For the loading of small drug molecules into the CaCO_3_ charged macromolecules can be entrapped in CaCO_3_ by co-precipitation and then provide additional attractive forces for positively charged drugs. There are numerous publications on polymer doping of CaCO_3_ and spontaneous drug loading to the polymer-doped carriers [122,127,128].

One of the most promising methods for the drug loading and delivery is fabrication of layer-by-layer assembled microcapsules composed of oppositely charged polyelectrolytes deposited on CaCO_3_ core [113,129]. This approach is suitable for varous compounds encapsulation, in particular, poorly soluble drugs.

The adsorption of macromolecules inside porous calcium carbonate particles is considered to be governed by electrostatic interactions on the CaCO_3_ surface and protein-protein interactions. Moreover, macromolecule adsorption onto solid-liquid interfaces can be determined by several other processes such as steric interactions due to the polymeric components at the core surface that extend into the surrounding aqueous phase. Changes in the state of hydration and rearrangements in the macromolecular structure may take place at regulation of molecules adsorption. Loading of macromolecules in porous CaCO_3_ particles is affected by their molecular weight due to the diffusion-limited permeation into the particles and by the affinity to the carbonate surface. By adjusting pH of the solutions the electrostatic interaction can be altered and, thus, adsorption-desorption process can be changed allowing controlling the amount of deposited macromolecules [130,131]. The layer-by-layer assembled microcapsules possess multiple functionalities such as multicomponent delivery and effective multienzyme catalysis [132].

Thus, drug-loaded CaCO_3_ particles are particularly suited for controlled release and delivery of a variety of biomolecules. These systems are expected to enhance the bioavailability of poorly absorbed drugs, entailing a lowering of the therapeutic dose. Some biological aspects of drug release are discussed further.

### 3.3. Drug Release

**Figure 6:**
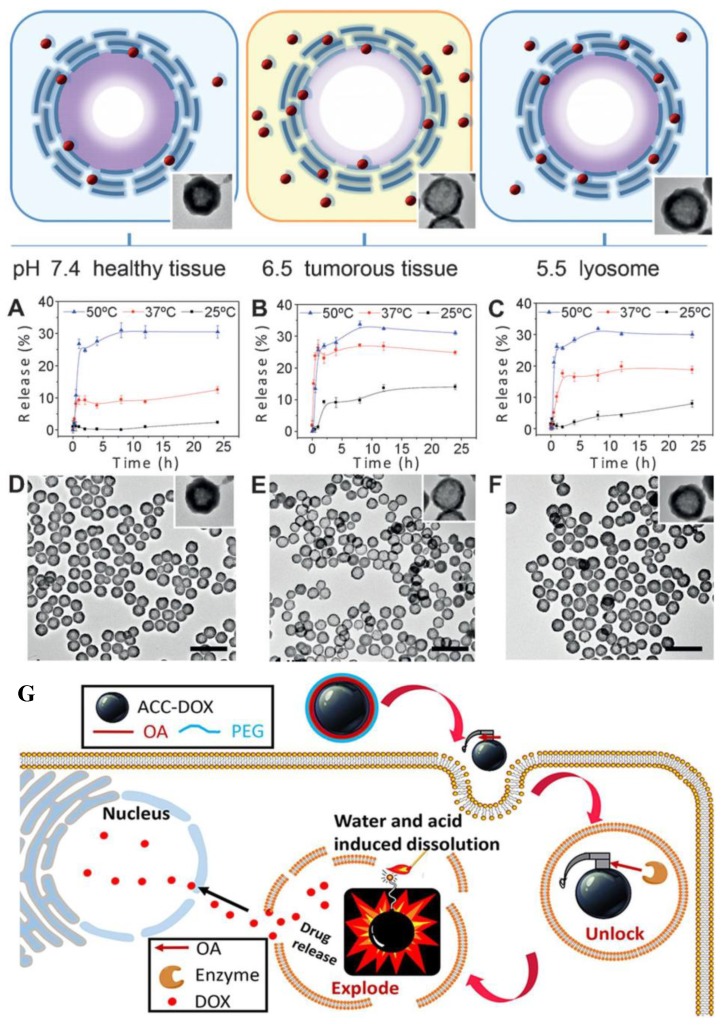
Schematic description of the pH-responsive drug release of ACC-DOX@silica nanoparticles. Reproduced with permission from [145]. Wiley Publishing Groups, 2012. Cumulative releases of doxorubicin (DOX) from ACC-DOX@silica suspensions in various aqueous buffers at (**A**) pH 7.4; (**B**) pH 6.5 and (**C**) pH 5.5 with different temperatures of 25, 37 and 50 °C. Representative TEM images of the suspensions under 37 °C at (**D**) pH 7.4; (**E**) pH 6.5 and (**F**) pH 5.5. Reproduced with permission from [145]. Wiley Publishing Groups, 2012. The scale bars are 500 nm. (**G**) Schematic illustration of assembly process and disassemble mechanism of PEG/OA-ACC-DOX within cancer cells. Reproduced with permission from [136]. Royal Society of Chemistry, 2017.

Since the vaterite particles are the least stable polymorph form of calcium carbonate, they tend to transform into calcite in aqueous environment following by the dissolution and crystallization [132]. The dissolution-precipitation process of the CaCO_3_ particles has been shown to allow controlling the drug release time. Parakhonskiy et al. have carried out experiments with porous vaterite particles of 400 nm in size loaded with rhodamine 6G and tetramethylrhodamine (TRITC)-dextran [133]. The low molecular weight rhodamine was releasing slowly during the first four days of the experiment in water and then the release rate increased dramatically induced by vaterite recrystallization. The high molecular weight cargo TRITC-dextran possessed a slow release rate, no recrystallization was observed during the first week of incubation. Thus, the polymer entrapped in CaCO_3_ acts as an inhibitor of the phase-transition. Similar effect has been shown by Lauth at el. The authors evaluated that the adsorption of bovine serum albumin (BSA) on the surface of CaCO_3_ crystals. Results indicated that BSA can slow down the rate of phase transformation of sub-micrometer vaterite particles [134]. Several examples can be found in the literature where different substances are used to regulate the crystallization of CaCO_3_ particles, such as starch [135], oleic acid [136], chitosan [137] and variety of proteins [138,139,140]. The release of the encapsulated drugs from CaCO_3_ porous structure has been shown to be sensitive to the environmental pH. The recrystallization of vaterite particles occurs more readily in acidic media pH < 5, but at neutral pH vaterite crystals are less soluble [133,141]. Release in this case is characterized by an initial burst release of the drug from CaCO_3_ particles followed by nearly constant release. Therefore, this makes vaterite particles beneficial for their use as pH responsive drug delivery systems, especially for the pH triggered release of the anticancer drugs in acidic environment of tumour and inside endo/lysosomal compartments of cells [115,142,143,144]. The release rate can be controlled, depending on the size of the particles and their pores [134]. Generally, it decreases with the decrease of the pore size. Capping the CaCO_3_ cores with multilayers allows tuning the release profile of the encapsulated drug as has also been shown in the literature [135,136]. Figure 6 depicts the typical release mechanism of anticancer drug loaded in CaCO_3_ particles at various pH. Schematic representation demonstrates a locking/unlocking mechanism of the burst drug release within cancer cells by using ACC carriers covered with an oleic acid (OA) shell and a polyethylene glycol (PEG) corona [136]. Figure 6E illustrates the above-mentioned statements and shows cumulative release profiles at different temperatures and pH values (7.4, 6.5, and 5.5) mimicking the microenvironment of healthy tissue, tumor tissue, and cellular lysosomes, respectively (Figure 6A–C) [135].

Adsorption of macromolecules allows preserving particle in vaterite polymorph, slowing down the process of recrystallization and decrease drug release rate [146]. Borodina et al. investigated the effect of the different coatings such as hyaluronic acid, poly _L_-lysine and surfactant (Tween 80) on loperamide release out of the CaCO_3_ carrier. The obtained results showed 10% of the release extension for microparticles modified with hyaluronic acid [147].

### 3.4. Delivery of Various Compounds In Vitro and In Vivo

The sustained release of drugs from the CaCO_3_ particles was confirmed both in vitro and in vivo experiments (Table 2). It possesses unique advantages due to their biocompatibility and the ability for loading different therapeutic agents. Studies show that CaCO_3_ nano/microparticles can be used as vectors to deliver various biological substances such as drugs [143,148], proteins [111,143,147,149], peptides [150,151] and genes [132,152]. Due to high biocompatibility, biodegradability, and pH sensitive features, vaterite and ACC show promising potential for the development of the drug delivery system for cancer treatment [143,148]. As it has been already mentioned, tumor tissues (pH 6.5–6.8), cell endosomes (pH 5.5–6.5), lysosomes (pH 4.5–5.5) and blood stream and normal tissues (pH 7.4) have different pH conditions [153]. Som et al. conducted in vivo experiments and showed that monodispersed CaCO_3_ nanoparticle can be used to intentionally modulate local pH and inhibit tumor growth. The authors evaluated that CaCO_3_ particle size affects alkalinization efficiency of the acidic pH of tumors. Dissolution of submicron particles of CaCO_3_ in vivo was shown to increase pH asymptotically to 7.4 [106].

Doxorubicin as anti-cancer drug is often employed to investigate the CaCO_3_ carrier system. Zhao et al. studied co-delivery of doxorubicin and Au–DNA nanoparticles by using CaCO_3_ carrier for drug and gene therapy [135,154]. It has been shown that the devolved vehicle exhibits high efficiency in nuclear invasion. The drug is observed to be widely distributed in the tumor tissue. Dong et al. applied the CaCO_3_ nanoparticles to deliver photosensitizer Hypocrellin B as a model hydrophobic anticancer drug. High toxicity of the particles has been detected in vitro by using breast cancer cell line (MCF-7) under light irradiation. Another water-insoluble anticancer drug, camptothecin, encapsulated in CaCO3 microspheres has been investigated by Qiu et al. [111].

On the basis of CaCO_3_ carriers multi-drug delivery systems consisted of doxorubicin and additional hydrophobic drug (paclitaxel or tariquidar) were developed by Wu et al. [137,155]. It has been shown that the dual drug loaded nanoparticles enhanced cell inhibitory effect, especially for drug resistant cancer cells. As it has been shown in the previous section, besides vaterite particles, ACC carrier can be applied for a burst release of its payload within cancer cells (Figure 6G). The pH-responsiveness of the ACC-DOX@silica nanoparticles after uptake by HeLa cells determines the sustained killing effects in the intracellular physiological environment as reported by Zhao et al. [145].

The antibacterial activity and pH-dependent drug release have been observed for CaCO_3_ microstructures containing tetracycline-entrapped polypeptides [150]. It has been shown that the encapsulated drug or the dye-conjugated peptide emits fluorescence suitable for optical imaging and detection, thereby making it a multitasking material.

CaCO_3_-based composite nanocarriers have potential for the peptide and protein pulmonary aerosol delivery and oral administration of insulin [156]. Liu et al. demonstrated that an improved hypoglycemic effect in vivo can be obtained compared with subcutaneous injection of insulin on a rat model [156].

Based on the given literature review, it can be concluded that CaCO_3_ porous carriers have a very prospective potential for site-specific drug delivery. However, calcium carbonate carriers also possess certain drawbacks, which should be taken into account. The synthesis procedure of calcium carbonate particles is in the most cases very sensitive and, thus, not always 100% reproducible. Slight variations of external temperature, speed of reaction mixing, purity of the precursors for the reaction can result in the size and shape changes of the calcium carbonate particles.

## 4. Calcium Phosphate Carriers

Calcium phosphate-based nanostructured materials are widely used in different applications in biology and medicine due to its biocompatibility and biodegradability [157,158]. An obvious advantage of calcium phosphate is its well-known osteoinductivity, this material is often used as a tissue implant and bone substitute [159,160,161]. Calcium phosphate is also often employed as drug delivery carrier of genetic materials [162,163], commercial drugs [164] and other bioactive molecules [165]. This material can be porous, what increases loading capacity of carriers and, thus, efficiency of delivery. Moreover, it is known that calcium phosphate dissolves at slightly acidic pH [166], what enables the possibility of controlled drug delivery into cells. After dissolution of the calcium phosphate carriers, ionic non-toxic constituents Ca^2+^ and PO_4_^3−^ remain, and this prevents particle accumulation and induces intracellular drug release [167]. All these features of calcium phosphate make this material attractive for the drug delivery applications.

### 4.1. Synthesis of Calcium Phosphate

**Figure 7:**
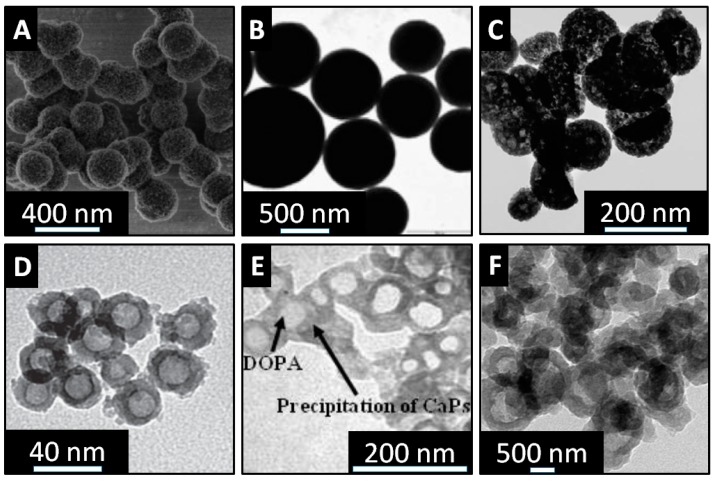
(**A**) SEM image of amorphous calcium phosphate particles synthesized by using CaCl_2_∙2H_2_O as the calcium source and ATP as both the phosphorous source and stabilizer by microwave-assisted hydrothermal method at 120 °C for 10 min. Reproduced with permission from [168]. Wiley Publishing Groups, 2013; (**B**) TEM images of calcium phosphate precipitates. Reproduced with permission from [177]. Bulletin of the Chemical Society of Japan, 2008; (**C**) TEM image of hollow calcium phosphate particles prepared using soybean lecithin, Na_2_ATP and CaCl_2_ by the microware-assisted hydrothermal method at 120 °C. Reproduced with permission from [171]. Royal Society of Chemistry, 2015; (**D**) TEM image of hollow calcium phosphate nanospheres. Reproduced with permission from [172]. Royal Society of Chemistry, 2012; (**E**) Transmission electron microscopy (TEM) image of calcium phosphate nanoshells synthesized with templates of DOPA. Reproduced with permission from [173]. Elsevier, 2012; (**F**) TEM image of calcium phosphate spheres. Reproduced with permission from [176]. IOPscience, 2009.

There are many different methods to produce calcium phosphate particles with different morphologies and sizes. For the drug delivery applications, porous calcium phosphate particles are of the particular relevance due to increased loading capacity. A traditional synthesis method of porous calcium phosphate nanosized materials involves mixing of water soluble salts of calcium and phosphate. With this technique, it is possible to control size, shape and crystallinity of particles by changing conditions of the precipitation reaction. For example, to obtain porous calcium phosphate spherical nanoparticles microwave assisted hydrothermal method is often used. This method involves adenosine 5-triphosphate disodium salt (ATP) as the phosphorus source and stabilizer (Figure 7A) [168]. As an example, Qi et al. synthesized porous calcium phosphate particles with an average diameter 260 nm by mixing calcium chloride dehydrate with ATP and further microwave treatment. The resulted nanoparticles showed good stability in aqueous solutions at different pH for more than 150 h, what is much longer than previously reported porous calcium phosphate particles [169]. Alternatively, porous calcium phosphate nanoparticles with different morphology and crystallinity can be prepared by changing the ratio of precursors. Kondori et al. showed that calcium phosphate particles can be synthesized by aging a mixture of calcium hydroxide and sodium triphosphate in the presence of hydrochloric acid [170]. To obtain differently shaped particles, authors varied aging temperatures from 100 to 150 °C, as well as amount of precursors in the reaction. Moreover, hollow calcium phosphate nanoparticles can be obtained with the templating method (Figure 7C) [171]. Different materials can be employed as templates. For example, Ding et al. used soybean lecithin to form calcium phosphate hollow particles. Another materials, which can be used as the template to form hollow calcium phosphate particles, are block copolymer micelles (Figure 7D) [172]. Bastakoti et al. used an anionic triblock copolymer (poly (styrene-acrylic acid-ethylene glycol) to form calcium phosphate hollow particles. This kind of template helps to overcome the problem of crystal overgrowth and allow the formation of nanosized hollow particles around 30 nm. Liposomes are also used as templates to form hollow calcium phosphate particles [173,174,175]. Yeo et al. employed 1,2-dioleoyl-sn-glycero-3 phosphate sodium salt (DOPA) and 1,2-dipalmitoyl-sn-glycero-3-phosphate sodium salt (DPPA) as templates due to their negative charged head group, which can help the deposition of calcium and phosphate ions around the liposomes. Resulted hollow particles were 64 and 104 nm, respectively (Figure 7E). Polymer complexes can be also employed as templates to form hollow calcium phosphate particles (Figure 7F) [176]. Zhang et al. prepared hollow calcium phosphate microspheres using chitosan-poly acrylic acid (CS-PAA) as the template. The formation mechanism of the hollow structure was based on the electrostatic interactions between chitosan (CS) and poly acrylic acid (PAA). The authors also showed that the size of CS-PAA spheres could be adjusted by changing the ratio and concentration of CS and PAA in the reaction.

Given the nature of encapsulated drugs, they can be entrapped into calcium phosphate particles or attached onto the particles surface. For the first scenario, drugs are usually caged into the particles matrix during their formation. This can be done with conventional co-precipitation reaction, drugs-calcium phosphate co-precipitates arise spontaneously in supersaturated solutions. Addition of the drugs during the synthesis of the particles can affect their physico-chemical properties. However, if prolonged intracellular release of drugs is needed, embedding of drugs into the interior of particles is more preferable. Alternatively, drugs to deliver can be attached onto the particles surface after calcium phosphate particle synthesis. In this case, however, drugs are less protected from the intra- and extracellular environment [178].

### 4.2. Release Efficiency

**Figure 8:**
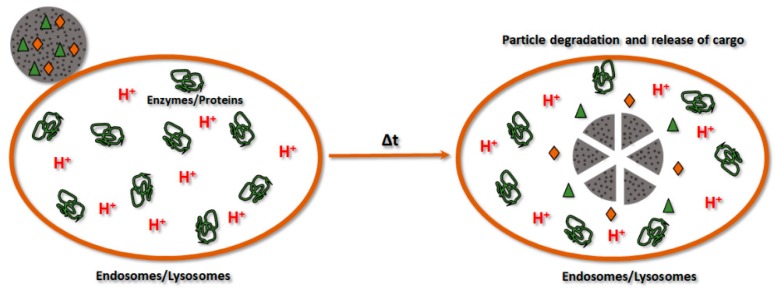
Schematic illustration of calcium phosphate particle degradation inside endosomal/lysosomal compartment (endosomal/lysosomal compartments are in orange color, enzymes/proteins are in green color, spherical calcium phosphate particle is in grey color, delivered cargoes are orange rhombs and green triangles).

Intracellular release of drugs from calcium phosphate particles can be usually controlled. Loaded drugs can be released in a prolonged way during the degradation of calcium phosphate particles in the acidic environment of the endosomal/lysosomal compartments (Figure 8) [179]. This stimuli-responsive behavior of calcium phosphate makes this material attractive for the drug delivery applications. Moreover, previous studies hypothesized cytosolic release of cargo delivered with calcium phosphate carriers. The dissolution of calcium phosphate particles induces the endosomal/lysosomal escape of delivered cargo due to the swelling and further bursting of these compartments and, consequently, intracellular release of cargo inside cells [150]. Interestingly, the release of cargo from calcium phosphate particles dispersed in slightly acidic pH can be controlled. Resent findings showed that cargo release from calcium phosphate particles, synthesized at different temperatures, is dependent on resorption of these particles [180]. Matsumoto et al. investigated release of proteins from calcium phosphate particles by immersion of particles in solutions at pH 4 and 7. The amount of released proteins from the particles immersed in the solution at pH 7 was significantly smaller than from solution at pH 4. Another way to control release of drugs with pH is employing of calcium phosphate as pore blocker material for mesoporous silica particles. Pores of this composite material can be loaded with drugs, whereas calcium phosphate coating can hold the encapsulated drugs under extracellular conditions. Inside cells calcium phosphate can be dissolved initiating intracellular drug release from the pores of mesoporous silica particles [56].

For the burst drug release from calcium phosphate particles, an external physical impact can be applied. For example, it has been shown that calcium phosphate nanospheres can be destroyed under the ultrasound treatment [181]. Cai et al. synthesized nanosized hollow calcium phosphate particles by a wet chemical reaction, and cetyltrimethylammonium bromide (CTAB) was used as a modifier. Ultrasound treatment induced the deconstruction of these hollow particles resulted in pin-like nanocrystallites of calcium phosphate.

### 4.3. In Vitro and In Vivo Delivery of Various Compounds with Calcium Phosphate Carriers

**Figure 9:**
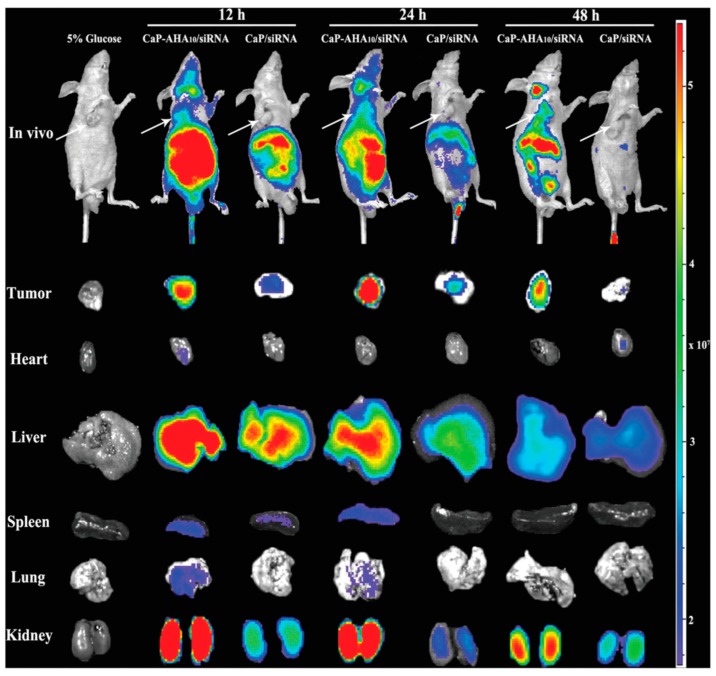
In vivo distribution of calcium phosphate particles loaded with siRNA observed by the in vivo imaging system. The nude mice bearing the A549 tumor was given intravenous injection via tail vein. Reproduced with permission from [189]. Royal Society of Chemistry, 2016.

Nucleic acids are the main compounds, which can be successfully delivered with calcium phosphate particles (Table 3) [182,183,184]. The first report related to the use of calcium phosphate as non-viral vector was made in 1973 by Graham and van der Eb [185]. Nowadays, in vitro transfection with calcium phosphate precipitates is a standard laboratory procedure. This method is cost-effective and easy-to-use; however, it suffers from some disadvantages, such as low reproducibility and poor transfection efficiency [186]. Pedraza et al. showed that transfection efficiency is reduced with aggregates of calcium phosphate particles loaded with DNA with sizes much higher than 200 nm. Removing the large agglomerates with filtration resulted in size of calcium phosphate particles much less than 200 nm and, thus, in an improved transfection of cells. More efforts were made to reduce the size of calcium phosphate particles. Calcium phosphate nanoparticles (less than 100 nm) showed improved biocompatibility and transfection efficiency in vitro [187,188]. Ultrasmall sized particles (20–50 nm) were synthesized by Cao et al. These particles were employed to deliver DNA into mesenchymal stem cells in vitro. Calcium phosphate particles had significantly lower toxicity in comparison to commercial transfection reagents, e.g., Lipofectamine 2000 [188]. Elangovan et al. successfully synthesized 30–50 nm calcium phosphate nanoparticles loaded with genes. These particles were then employed to transfect fibroblasts in vitro. Interestingly, transfection studies revealed a higher and longer lasting transfection up to 96 h. Qiu et al. showed that high accumulation of calcium phosphate particles loaded with small interfering RNA (siRNA) in tumor led to a significant tumor growth inhibition with a specific gene silencing effect in vivo [189]. In vivo distribution of these particles in an A549-xenografted mouse showed significant accumulation in the liver after first 12 h with further excretion by renal metabolism after 48 h (Figure 9). Another study showed anti-inflammatory effect of calcium phosphate particles loaded with siRNA in vivo. siRNA was directed against tumornekrosefaktor (TNF-α), keratinocide-derived cytokine (KC) or interferon gammainduced protein (IP-10) to mice suffering from induced colonic inflammation [190]. Multiple therapeutic agents can be delivered with the carriers based on calcium phosphate. Zhou et al. demonstrated successful co-delivery of inhibitors for microRNA221 and microRNA-222 (miRi-221/222) and paclitaxel (pac) [191]. As the result, compounds were delivered into MDA-MB-231 cells inhibiting the proliferative mechanism of miR-221/222, cyclin-dependent kinase inhibitor (p27^Kip1^) and tissue inhibitor of metalloproteinase 3 (TIMP3).

To increase the local drug concentrations in the desired areas and minimize the risks of the systemic toxicity, capsulate drug delivery systems based on calcium phosphate have been developed [192]. Xu et al. successfully encapsulated hydrophobic drug indomethacin into the cavity of calcium phosphate particles. Another toxic drugs, like cisplatin [193], were encapsulated into the cavity of the liposomes and at the same time liposomes were employed as templates for biocompatible calcium phosphate. The architecture of hollow calcium phosphate spheres benefits due to improved loading capacity and pH-sensitivity. Moreover, it has been reported that calcium phosphate coating reduces drug release rate in comparison to the uncoated liposomes enabling prolonged drug release [192]. Hydrophobic drugs can be also loaded within the calcium phosphate shell with the same templating method [173,192].

Verma et al. reported for the first time the use of Vitamin B12 conjugated with calcium phosphate particles loaded with insulin for the enhance the absorption of the particles by multiple pathways apart from the gastric intrinsic factor (IF) receptor mediated endocytosis. These delivery systems can be effectively applied for the oral administration. Photodynamic therapy of tumor in vivo was also realized with calcium phosphate particles loaded with the photosensitizer Temoporfin. These particles were additionally functionalized with peptides for targeted delivery into endothelial cells. After 2 days of particle treatment of mice, apoptosis in the tumor was detected [194]. Moreover, tumor vascularization was destroyed what makes calcium phosphate particles very promising drug delivery system.

Despite this, calcium phosphate have some limitations as drug delivery platform. First of all, as it has been mentioned above, calcium phosphate carriers are mostly used to deliver nucleic acids into cells. However, transfection efficiency of these carriers is still poor in comparison with commercial reagents (e.g., Lipofectamine) or viral carriers [188]. Moreover, encapsulated materials are limited to solubility in water or organic solvent [195]. Nevertheless, calcium phosphate nanoparticles are commonly accepted to be a nontoxic, biocompatible, and resorbable material and, thus, suits as nanocarrier for the drug delivery applications [196].

## 5. Concluding Remarks and Future Research Directions

As it has been reported above, the porous inorganic materials such as mesoporous silicas, calcium phosphates and calcium carbonates were widely applied as carriers in the drug delivery applications. These systems possess multiple features such as high loading of bioactive substances, in vivo navigation and visualization, remote release of cargo by internal and external stimuli. The morphological and surface properties of silicas, calcium carbonates and calcium phosphates allows to perform the organic functionalization in order to develop novel efficient hybrid carriers with enhanced multimodal properties. Moreover, there are a lot of possibilities for the in situ co-encapsulation of various bioactive compounds (drug, gene, protein, fluorescent molecule, etc.).

However, these materials can also possess certain drawbacks, which are associated with their safety and stability. Although these materials are considered to be non-toxic, it is always important to control the concentration of employed particles in order to avoid adverse effects. Moreover, when working with biological fluids (cell culture medium, blood plasma etc.) more factors concerning stability of particles should be considered. For instance, organic compounds in biological fluids tend to bind to surface of drug carriers, forming so-called corona around the particles. This corona can significantly change physico-chemical properties of particles, resulting in particles aggregation. Therefore, they should be characterized not only in aqueous solutions, but also in biological fluids.

In general, it can be concluded that the silica-based carriers are mostly used for the delivery of antitumor drugs and fluorescent dyes while calcium phosphate carriers are more appropriate for the delivery of genetic materials (e.g., DNA, microRNA, siRNA) and osteogenic factors (e.g., dexamethasone) for the bone tissue engineering. The calcium carbonate particles are mostly applied for the delivery of anticancer drugs (doxorubicin, vincristine, etc.), osteogenic factors and protein encapsulation.

The sol-gel chemistry is used for the fabrication of porous silica-based carriers. As for the silica-based carriers, combination of sol-gel chemistry, organic functionalization, and surface decoration with inorganic nanoparticles is a key principle for creation of nanostructured hybrid silica carriers with fundamentally new features. The introduction of organic components into the silica frameworks (e.g., the incorporation of disulfide bonds) leads to an interesting responsive degradation behavior and drug release. However, it is still difficult to prepare the organic-functionalized silica carriers with well-defined structures. Therefore, the fabrication of porous organic-inorganic silica carriers with high stability is highly desirable.

The preparation of calcium carbonate carriers with loaded drugs is mainly based on emulsion techniques and chemical precipitation. Usually pH-sensitive properties of calcium carbonate are used for release of anticancer drugs. Since the pH of tumor tissues bellow 6.5, it is still important to develop targeted drug carriers based on calcium carbonate nanoparticles. To increase the targeted delivery, the nanostructures of calcium carbonate should be functionalized with targeting agents. Moreover, there are some problems, such as incomplete in vivo release of drugs or/and incomplete degradation, that should be considered to improve the drug delivery properties of calcium carbonate.

The use of calcium phosphate carriers for the drug delivery application requires specific functionalities, which are difficult to develop. However, recent progress in nanofabrication technologies opens possibilities to fabricate nanostructures of calcium phosphate with diverse morphology, size and composition. At present, we may highlight that calcium phosphate is mostly used for co-precipitation with DNA to perform gene delivery. The main disadvantages of the calcium phosphate gene transfer system are not always sufficient transfection efficiency and poor reproducibility compared with other non-viral carriers (e.g., Lipofectamine 2000). Although a lot of new methods based on the calcium-phosphate-DNA precipitation have been developed to enhance transfection efficiency, it is vital to improve the transfection efficiency using new synthetic approaches, for example, combining calcium phosphate carriers with other materials. The combination of porous inorganic carriers with organic/inorganic nanomaterials creates a synergetic effect for drug delivery applications and diagnostics.

In the near future, scientists should pay more attention to in vivo biosafety and degradation of drug carriers. Even though a great amount of different porous carriers was developed, most of them not introduced into preclinical studies. This is mostly because used for the functionalization of porous carriers compounds and carriers itself are not always approved by the medical committee. Moreover, more experimental data are needed to study the mechanism of the in vivo degradation of carriers and their removal from the body. The clear understanding of how the composition/structure of porous drug carriers affects the in vivo safety and degradation could pave the way for the improved treatment of diseases with higher biosafety.

## Figures and Tables

**Table 1:** Recently studied silica-based drug delivery systems.

Synthesis Method	Particle Size	Active Drug	In Vitro/In Vivo Results	Ref.
Sol-gel method Mix CTAB with NaOH solution. Add TEOS at 80 °C to the surfactant solution. Calcination was performed at 550 °C for 6 h.	100 nm	Doxorubicin	The folate-receptor-mediated targeted delivery was proved by qualitative confocal laser scanning microscopy (CLSM) measurement. Cellular uptake of the doxorubicin-loaded nanoparticles in folate receptor-overexpressing HeLa cells was detected to be much higher than that in non-folate receptor-overexpressing adenocarcinomic human alveolar basal epithelial cells (A549) cells.	[74]
Sol-gel method Mix CTAB with NaOH solution. Add TEOS at 80 °C to the surfactant solution. Collect precipitate, wash with water and methanol, dry with vacuum. Resuspend particles in methanol, HCl and stir at 60 °C for 48 h.	200 nm	Doxorubicin	The human liver cancer cell line (HepG2) cells viability with drug-loaded ZnO@MSN decreased below 50% at a concentration of 50 µg/mL, which is two times lower than that viability of cells incubated with free doxorubicin.	[55]
Template method Disperse polystyrene nanoparticles functionalized with sulfonic groups in ethanol and ammonia. Add TEOS and stir under 50 °C for 3 h. Soak polystyrene nanoparticles with tetrahydrofuran.	200 nm	Doxorubicin	Synthesized GSH-responsive hollow silica nanoparticles showed near 3-fold higher doxorubicin release at pH 5.0 than at pH 7.4 without the presence of GSH. Qualitative CLSM observations confirmed that doxorubicin-loaded nanocarriers were internalized and the drug was released to reach cell nuclei within 24 h.	[77]
Sol-gel method Mix CTAB with NaOH solution. Add TEOS at 80 °C to the surfactant solution. To remove CTAB, reflux in HCl and methanol for 16 h.	150 nm	Doxorubicin	Synthesized GSH responsive silica nanoparticles capped with peptide incubated separately with avβ3-integrin-positive tumor cell line (U87 MG) and avβ3-integrin-negative cell line (COS 7) at a doxorubicin concentration of 2 µg mL^−1^ possessed 1.7-fold higher U87 MG cellular uptake than that of silica nanoparticles without peptide. There was very little difference between the carriers in COS 7.	[60]
Sol-gel method Mix CTAB with NaOH solution. Add TEOS at 80 °C to the surfactant solution. To remove CTAB, add ethanol and HCl at 60 °C for 24 h.	40 nm	Doxorubicin	Hyaluronic acid modified silica-based nanocarriers showed redox-responsive drug release and high drug loading (14%). The developed carriers were internalized in 2-fold higher amounts in HeLa cells than in LO2 cells.	[61]
Sol-gel method Mix CTAB with NaOH solution. Add TEOS at 80 °C to the surfactant solution. Calcination was performed at 550 °C for 5 h.	100 nm	Doxorubicin and dye	GSH-responsive PEG-capped silica-based particles were used to deliver safranin O and doxorubicin in a controlled manner in vitro, achieving 90% of the maximum release of the entrapped drug in less than 1 h. The results showed that the PEG-capped systems were closed at low GSH concentrations. The cargo was released/delivered when the concentration of GSH increased.	[63]
Sol-gel method Mix CTAB with NaOH solution. Add TEOS at 80 °C to the surfactant solution. Calcination was performed at 550 °C for 5 h.	150 nm	Cisplatin and doxorubicin	Polymer-gatekeeper mesoporous silica nanoparticles were synthesized by noncovalent capping of the pores of drug-loaded nanocontainers with disulfide cross-linkable polymers. By varying crosslinking density from 19% to 83%, the drug release was decreased almost in 2-fold.	[64]
Template method Add TEOS to the polystyrene latex nanoparticles. Calcination was performed at 600 °C to remove polystyrene nanoparticles.	150 nm	Doxorubicin	The in vitro experiments indicated that the silica-based nanocarriers modified by near infrared (NIR)-light responsive polymers had a considerable drug loading efficiency of more than 70%. A significant number of drug molecules (>50%) could be released from the nanocarriers upon NIR-light irradiation.	[69]
Mix CTAB with NaOH solution. Add TEOS and APTES at 80 °C to the surfactant solution. Wash with ethanol.	100 nm	Doxorubicin	A novel multifunctional envelope-type mesoporous silica nanoparticle system was used for drug delivery and magnetic resonance imaging (MRI) in vivo. The doxorubicin release was markedly increased under acidic conditions; more than 60% and 90% of the drug were released at pH 5.0 and pH 2.0, respectively.	[87]
Sol-gel method Mix CTAB with NaOH solution. Add TEOS at 80 °C to the surfactant solution. To remove CTAB, reflux in HCl and methanol for 24 h.	100 nm	FITC dye, (S)–(+)– camptothecin	The cumulative drug release from the synthesized MSN@Fe_3_O_4_ nanocarriers was increased from 0.2% to about 21.9% over a 5 min magnetic stimulus. Obtained carriers showed T_2_-type MR contrast enhancement for cell or molecular imaging.	[88]

**Table 2:** Recently studied CaCO_3_ drug delivery systems.

Synthesis Method	Particle Size	Active Drug	In Vitro/In Vivo Results	Ref.
CO_2_ diffusion through CaCl_2_ in EtOH	100 nm	No drug	Resulted carriers modulated local pH and repeated daily administration of nano-CaCO_3_ inhibit tumor growth up to 2-fold in comparison with control. Efficient alkalization of the acidic pH of tumors depended on the particle size.	[106]
CaCl_2_ + NaHCO_3_ 1:5 DI H_2_O:polyethylene glycol Mix for 5 min	20 nm			
CaCl_2_ + NaHCO_3_ 1:5 DI H_2_O:ethylene glycol Mix for 30 min	300 nm			
NH_4_HCO_3_ vapor diffusion through CaCl_2_ in EtOH oleic acid stabilizer and polyethylene glycol corona	600 nm	Doxorubicin	ACC particles with an oleic acid shell and a polyethylene glycol (PEG) corona showed retarded drug release profile with merely 14% of the drug being released after 24 h, decreased pH value up to 5.5 did not drastically increase the drug release, revealing that the drug locking effect was well realized.	[136]
CaCl_2_ + NaHCO_3_ water:gelatin:ethylene glycol Mix for 2 h	250 nm	photosensitizer Hypocrellin B	The cellular internalization of hybrid nanoparticles modified by cross-linked hyaluronic acid by MCF-7 cells overexpressing cell-surface glycoprotein (CD44 receptor) has been enhanced from 2.2% to 85%, endowing the nanoparticles with targeting functionality.	[153]
CaCl_2_ + NaHCO_3_ starch solution Mix for 10 min	800 nm	Doxorubicin, Au–DNA	Qualitative CLSM observations showed efficient intracellular delivery of doxorubicin by the CaCO_3_ carriers and especially into the nuclei of A549 and HeLa cells.	[135]
Polypeptide mediated mineralization from CaCl_2_ + (NH_4_)_2_CO_3_	500–1000 nm	Tetracycline	The IC50 (half maximal inhibitory concentration) values for various cell lines indicated relatively greater cell inhibition (from 1.8 to 8 fold) in the case of all the three cancer cell lines (A549, epithelial human breast cancer cell line (MDA-MB-231) and HeLa) in comparison to the normal cells.	[150]
CaCl_2_ + NaHCO_3_ ethylene glycol (EG), gelatin Mix for 2 h	230 nm	Doxorubicin	Vaterite nanoparticles embedded with folic acid containing doxorubicin exhibited 2-fold higher cytotoxicity to MCF-7 cells compared with that of drug-loaded vaterite particle and free drug at concentrations of 0.02 and 0.04 mg/mL.	[154]
Spray Drying (NH4)_2_CO_3_ + Ca(OH)_2_ Hyaluronate polysaccharide	4600 nm	Salmon calcitonin, alpha-1-antitrypsin protein	The bioavailability of salmon calcitonin after aerosol delivery as peptide-loaded composite microparticles to rats was 4-times higher than that of salmon calcitonin solution.	[151]
Starch solution and starch-octanoic acid were set as templates to prepare CaCO_3_ nanoparticles from CaCl_2_ + NaHCO_3_ Mix for 30 min	400–500 nm	Doxorubicin	The IC50 of drug-loaded nanoparticles synthesized using 0.0625% starch-octanoic was almost 24-fold higher than that of DOX·HCl indicating higher A549 cellular uptake and faster drug release at the acidic pH.	[148]
CaCl_2_ + NaHCO_3_ 1–2% hyaluronic acid	150–350 nm	Insulin	An effective hypoglycemic effect was obtained in vivo compared with subcutaneous injection of insulin. After oral administration of insulin-loaded CaCO_3_ the blood glucose level is decreased 2-times slower for 3 h than that of with using insulin solution.	[156]
CaCl_2_ + NaHCO_3_ sodium alginate	200 nm	Doxorubicin, paclitaxel	Qualitative CLSM observations showed that the dual drug loaded nanoparticles exhibited significantly enhanced cell uptake and nuclear localization as compared with the single drug loaded nanoparticles.	[155]
CaCl_2_ + Na_2_CO_3_ chitosan	>200 nm	Doxorubicin, P-glycoprotein inhibitor (tariquidar)	Enhanced multidrug-resistant breast cancer (MCF-7) cells uptake and nuclear localization were observed qualitatively for the drug-loaded nanoparticles by CLSM. Drugs co-delivery systems demonstrated near 2-fold higher cell inhibition rates compared with doxorubicin delivery systems.	[137]

**Table 3:** Recently studied calcium phosphate drug delivery systems.

Synthesis Method	Particle Size	Active Drug	In Vitro/In Vivo Results	Ref.
Water-in-oil micro-emulsion method CaCl_2_ (in Cyclohexane/Igepal) + Na_2_HPO_4_ (pH = 9.0) in oil Mix for 20 min	40 nm	siRNA	A 40-fold improved silence activity compared to the previous analogous formulations. Reported nanoparticle vehicles effectively delivered siRNA to a solid tumor in a xenograft model in vitro and in vivo.	[182]
CaCl_2_ + Na_3_Cit + Na_2_HPO_4_ at pH 8.5 Mix for 5, 10, 20, 60 min at 37 °C	20–50 nm	miRNA	Calcium phosphate nanoparticles efficiently internalized into cardiomyocytes. Dose-response graphs are given. The nanoparticles did not show promoting toxicity or interfering with any functional properties. Nanoparticles successfully encapsulated synthetic miRNAs, which were efficiently delivered into cardiac cells in vitro and in vivo.	[183]
CaCl_2_ (in Cyclohexane/Igepal) + Na_2_HPO_4_ (pH = 9.0) in CHCl_3_	52–56 nm	DNA	Resulted calcium phosphate carriers showed multifunctional features. PEGylation of these carriers enabled delivery to hepatocytes in vivo. Co-delivery of cationic peptides CR8C supported extensive nuclear translocation of DNA in post-mitotic cells. Monocyclic CR8C significantly enhanced in vivo gene expression over 10-fold more than linear CR8C. Carriers had improved stability and protecte DNA from degradation, though 100-fold lower in gene expression was detected, the developed system presents a greatly decreased invasiveness in its application.	[184]
CaCl_2_ in bis (2-ethylhexyl) sulphosuccinate (in hexane) + Na_2_HPO_4_ in bis (2-ethylhexyl) sulphosuccinate Mix overnight at 4 °C	100–120 nm	DNA	Resulted carriers showed the transfection efficiency of 3% higher than that from the commercial transfecting reagent Polyfect.	[197]
Ca(NO_3_)_2_ + (NH_4_)_3_PO_4_ precipitated using a Harvard 22 syringe pump	20–150 nm	DNA	Effective transfection with calcium phosphate particles in different cell types was demonstrated.	[186]
CaCl_2_ (in Cyclohexane/Igepal) + Na_2_HPO_4_ (in Tris-HCl/Cyclohexane/Igepal) Mix for 10 min at 4 °C	20–50 nm	DNA	Resulted DNA carriers showed 5.7% lower cytotoxicity than commercial reagent Lipofectamine 2000. It has been demonstrated that calcium phosphate nanoparticles can be developed into an effective alternative as a non-viral gene delivery system.	[188]
CaCl_2_ + H_24_Na_3_O_16_P (in NaCl/KCl/dextrose/4-(2-hydroxyethyl)-1-piperazineethanesulfonic acid) Syringe pump	30–50 nm	DNA and growth factors	Synthesized calcium phosphate particles showed higher levels of biocompatibility (between 84% and 95% of mouse embryo (NIH-3T3) fibroblasts viability) and transfection efficiency into fibroblasts in vitro. Enzyme-linked Immunosorbent Assay (ELISA) test showed 3-fold higher platelet-derived (PDGF-B) growth factor expression in NIH-3T3 fibroblast cell line upon administration of nanocarriers than that of other applied substances.	[187]
CaCl_2_ + Alendronate-hyaluronic acid conjugate (in HEPES)	170 nm	siRNA	Resulted calcium phosphate carriers showed effectively delivered siRNA in the A549 cells and contributed to the gene silencing (with the efficiency of about 40%) in vivo and in vitro.	[189]
C_6_H_12_CaO_6_ + (NH_4_)_2_HPO_4_	150 nm	siRNA	Particles exhibited rapid cellular uptake, almost no toxicity, and reduced gene expression of approximately 50% compared to the controls. A specific knockdown of target genes at the site of inflammation was achieved.	[190]
CaCl_2_ (in Igepal/Cyclohexane) + Na_2_HPO_4_ (in Igepal/Cyclohexane/DOPA in chloroform)	100 nm	Paclitaxel and miRNA-221/222 inhibitors	Carriers loaded with multiple drugs simultaneously delivered paclitaxel and miRNA-221/222 inhibitors to their intracellular targets, leading to inhibit proliferative mechanisms of mRNA-221/222 with further enhancing the therapeutic efficacy of paclitaxel. It was demonstrated that the co-delivery nanocarrier system had 80% efficiency of tumor cell suppression when compared to free paclitaxel or delivering nanocarrier with a single drug (i.e., miRi only or paclitaxel only).	[191]
Ca(NO_3_)_2_ + K_2_HPO_4_ Stir for 1 h	129 nm	Cisplatin	Synthesized calcium phosphate nanoparticles were non-toxic. Drug-loaded nanoparticles showed comparable cytotoxicity to free drug in an in vitro cell proliferation assay using the cisplatin-resistant human ovarian carcinoma (A2780cis) cell line. Negatively charged drug nanoconjugates are unable to overcome drug resistance and had the 4-fold increase in IC50 value as compared to the free drug.	[193]
C_6_H_10_CaO_6_ + (NH_4_)_2_HPO_4_ Stir for 20 min	200 nm	Temoporfin, cyclic Arginine-Glycine-Aspartic acid-Phenylalanine-Lysine (RGDfK) peptide, fluorescent dye	Efficient drug delivery resulted in 2 times decrease in tumor vascularization in 1 week of treatment in vivo. Synthesized carriers combined diagnostic imaging, tumor targeting and drug delivery properties.	[194]
